# A simple and scalable hydrogel-based system for culturing protein-producing cells

**DOI:** 10.1371/journal.pone.0190364

**Published:** 2018-01-02

**Authors:** Qiang Li, Qiaofeng Wang, Ou Wang, Kaifeng Shao, Haishuang Lin, Yuguo Lei

**Affiliations:** 1 Department of Chemical and Biomolecular Engineering, University of Nebraska, Lincoln, Nebraska, United States of America; 2 Biomedical Engineering Program, University of Nebraska, Lincoln, Nebraska, United States of America; 3 Shanghai East Hospital, Tongji University School of Medicine, Shanghai, China; 4 Shanghai Advanced Research Institute, Chinese Academy of Sciences, Shanghai, China; 5 Mary and Dick Holland Regenerative Medicine Program, University of Nebraska Medical Center, Omaha, Nebraska, United States of America; 6 Fred & Pamela Buffett Cancer Center, University of Nebraska Medical Center, Omaha, Nebraska, United States of America; Xi'an Jiaotong University School of Medicine, CHINA

## Abstract

Recombinant protein therapeutics have become important components of the modern medicine. Majority of them are produced with mammalian cells that are cultured either through adherent culturing, in which cells are cultured on substrates, or suspension culturing, in which cells are suspended and cultured in agitated cell culture medium in a culture vessel. The adherent cell culturing method is limited by its low yield. In suspension culturing, cells need extensive genetic manipulation to grow as single cells at high density, which is time- and labor-consuming. Here, we report a new method, which utilizes a thermoreversible hydrogel as the scaffold for culturing protein-expressing cells. The hydrogel scaffolds not only provide 3D spaces for the cells, but also act as physical barriers to prevent excessive cellular agglomeration and protect cells from the hydrodynamic stresses. As a result, cells can grow at high viability, high growth rate, and extremely high yield even without genetic manipulations. The cell yield in the hydrogels is around 20 times of the suspension culturing. In addition, the protein productivity per cell per day in the hydrogel is higher than the adherent culturing method. This new method is simple, scalable and defined. It will be of great value for both the research laboratories and pharmaceutical industry for producing proteins.

## Introduction

Recombinant protein therapeutics have become important components of the modern medicine [1,2]. Hundreds of recombinant protein therapeutics have been approved by the United States Food and Drug Administration (FDA) [3,4]. Majority of them are produced with mammalian cells in culture [2], such as Chinese Hamster Ovary (CHO) cells [5], human embryo kidney (HEK293) cells [6] and PER.C6 cells [7]. These protein-producing mammalian cells are cultured with two major methods: adherent cell culturing, in which cells are cultured on substrates such as roller bottles [8] or microcarriers [9–11], and suspension culturing, in which cells are suspended and cultured in agitated cell culture medium in a culture vessel such as stirred-tank bioreactors [2,12]. The adherent cell culturing method has limitations including anchor-dependent requirement, low yielding, and batch-to-batch variations that make it difficult to culture cells in large scales [2,12]. As a result, suspension culturing is currently preferred for large-scale cell culturing and protein production [2,12].

Among the many mammalian cell types, CHO cells are the most used for protein production for a few reasons [2,12]. First, CHO cells can be engineered to resist the hydrodynamic stresses generated by the agitation in suspension culturing and grow at high density as single cells (e.g. up to 2x10^7^ cells/mL) [2]. Second, CHO cells can be adapted to grow in serum-free medium [13,14]. Serum products are highly unwanted for therapeutic protein production [12]. Though having these advantages, developing a high-quality CHO cell line for protein production is time- and labor-consuming [3]. In a typical cell line development, CHO cells are transfected with a plasmid vector that encodes the therapeutic protein. Through a series of selections under gradually increased selection pressure, clones with high survival rate, high growth rate and high protein productivity (i.e. the amount of protein produced per cell per day) are selected for protein production [1,15]. The process takes 6 to 12 months. Additionally, these selected clones gradually lose their productivity during the culture [1,2,15]. Other protein-producing mammalian cell types cannot be engineered and selected as easily as CHO cells to resist the hydrodynamic stresses. As a result, they either cannot grow as single cells or cannot grow at high density as single cells in suspension culturing [1,2].

**Fig 1:**
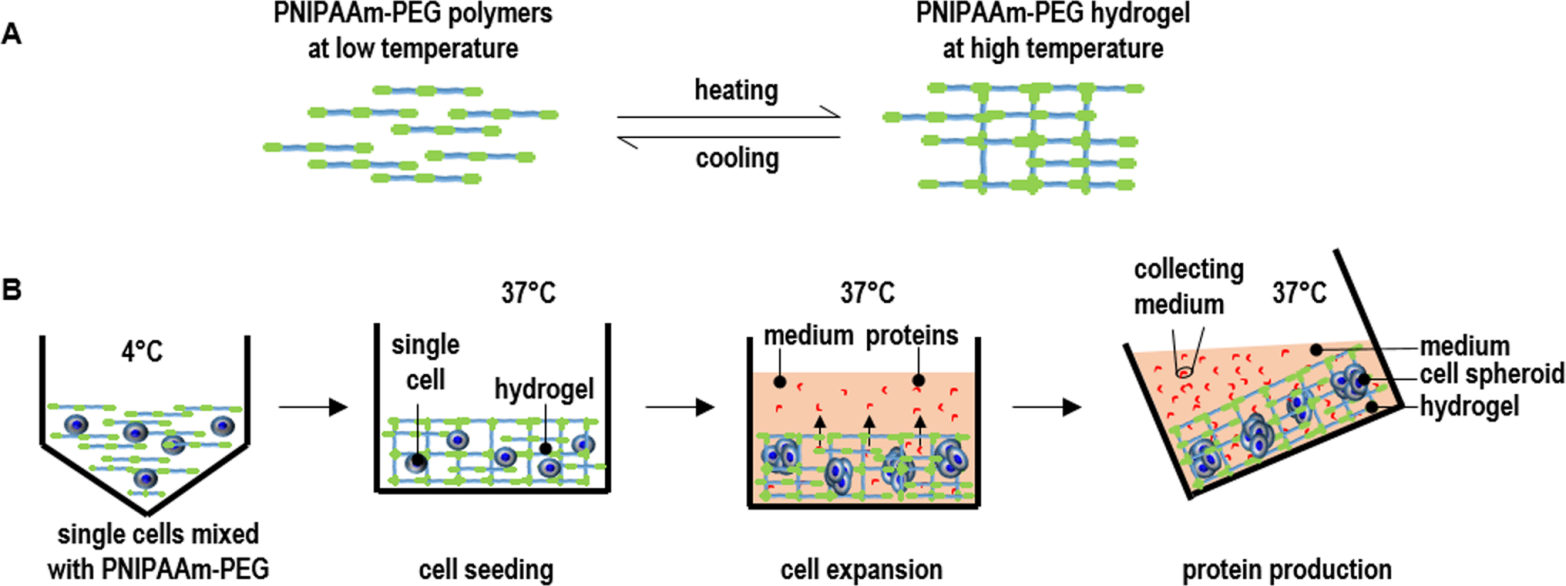
Overview of culturing protein-producing cells in three dimensional (3D) thermoreversible PNIPAAm-PEG hydrogels. (A) The PNIPAAm-PEG polymers are soluble in water at low temperature (e.g. 4°C). They associate to form a network at high temperature (e.g. 37°C), resulting in a hydrogel. When the temperature is reduced (e.g. to 4°C), the polymers become soluble again and the hydrogel is liquefied. (B) To culture cells, single cells are mixed with 10% PNIPAAm-PEG solution at low temperature and casted onto a tissue culture plate, and then incubated at room temperature or 37°C for 5 minutes to form an elastic hydrogel before adding the warm medium. The single cells clonally expand into uniform spheroids within days. Proteins secreted from the cells can diffuse through the hydrogel into the medium. The conditioned medium is collected for purifying the proteins.

We hypothesize that culture methods that can provide the protein-expressing mammalian cells a hydrodynamic stress-free environment will be of high value for therapeutic protein production. Without the hydrodynamic stresses, mammalian cells may be able to grow at high density with high productivity even without extensive genetic engineering and selection. Here, we report a new method, which utilizes a thermoreversible hydrogel made from PNIPAAm-PEG polymers as the scaffold for culturing protein-expressing cells. The aqueous solution of PNIPAAm-PEG polymers (Mebiol^®^ Gel, Cosmo Bio, USA) is liquid at low temperatures (e.g. below 4°C) (Fig 1A). The polymers in the solution associate through hydrophobic interactions to form an elastic hydrogel at high temperature (e.g. above 22°C) (Fig 1A). The hydrogel can be readily liquefied when the temperature is reduced (e.g. below 4°C) (Fig 1A). To culture cells, single cells are mixed with the 10% PNIPAAm-PEG solution at low temperature that is subsequently casted on the tissue culture plates at room temperature to form a thin layer of hydrogel before adding warm medium for growing cells (Fig 1B). The cell-mixed PNIPAAm-PEG solution can also be extruded into hydrogel fibers that can be suspended in cell culture medium. Within the hydrogel scaffold, single cells clonally expand into uniform spheroids within days (Fig 1B). The proteins secreted from the cells can freely diffuse through the hydrogel into the medium that is frequently collected (Fig 1B). This new method is simple and scalable. In addition, the PNIPAAm-PEG polymers are synthetic, defined, non-toxic to cells and available in large scales [16–18]. This new method can be used by both the research laboratories and pharmaceutical industry for producing proteins.

In this report, we used the murine L cells expressing the human Wnt3A proteins (i.e. L-Wnt-3a-cells) [19,20] as a model system to demonstrate this novel culture method. Wnt3A proteins, which play key roles in regulating stem cell self-renewal and differentiation, embryonic development, adult tissue homeostasis and cancer progression, have high therapeutic potential for treating many diseases [19,21–24]. Wnt3A proteins are lipid-modified and have very limited solubility in aqueous solution (e.g. maximal solubility is around 200 ng/mL) [19,21–24]. A method that can culture the Wnt3A-expressing cells at high density, while allow continuously collecting the conditioned medium in an easy manner is of great value for the cost-effective production of Wnt3A proteins. To show the superiority of the new method, we also cultured the L-Wnt-3a-cells with the conventional adherent and suspension culturing as comparison.

## Materials and methods

### Materials

L-Wnt-3a-cells (ATCC^®^ CRL-2647™) were acquired from ATCC. Cell culture reagents and their supplies: PNIPAAm-PEG polymers (Mebiol^®^ Gel, Cosmo Bio, USA); Trypsin-EDTA 0.05% (Invitrogen); Trypsin inhibitor (SIGMA); LIVE/DEAD^®^ Cell Viability staining (Invitrogen). Luciferase assay kit (Biovision, K801-200). DMEM (GE Healthcare Life Sciences); FBS (Atlanta biologicals); Recombinant human Wnt3A protein (R&D systems). Trypan blue solution was obtained from Sigma-Aldrich.

### Two-dimensional (2D) adherent culturing

L-Wnt-3a-cells were cultured in 6-well plate with DMEM medium supplemented with 10% FBS. The medium was changed daily, and cells were passaged every five days. Briefly, confluent cells were treated with 0.05% trypsin at 37 ^o^C for 3 minutes and dissociated into single cells using a pipette. 1x10^5^ cells were plated in one well of 6-well plate.

### Three-dimensional (3D) suspension culturing

L-Wnt-3a-cells were suspended in DMEM medium supplemented with 10% FBS in low attachment 6-well plates. The plates were shaken at 60 rotations per minute (rpm). The medium was changed daily. For the medium change, cells were transferred into 15 mL tube and centrifuged 5 minutes at 200 g. The medium was discarded before adding fresh medium.

### Culturing L-Wnt-3a-cells in 3D PNIPAAm-PEG hydrogels

Single L-Wnt-3a-cells were mixed with 10% PNIPAAm-PEG solution at 4°C and casted on a 12-well plate, then incubated at room temperature or 37°C for 5 minutes to form a hydrogel before adding warm DMEM medium supplemented with 10% FBS. The medium was changed daily. To passage cells, ice-cold PBS was added for 2 minutes to dissolve the hydrogel. Cell masses were collected by centrifuging at 100 g for 3 minutes and treated with 0.05% trypsin at 37°C for 5 minutes. Cells were dissociated into single cells with pipettes for the next passage. Cell viability was qualitatively assessed with live/dead cell staining according to the product manual (life technology). To quantify the viability, cells were stained with trypan blue and % of live cells were measured with a cell counter (TC20™, Bio-Rad).

### Qualitative detecting Wnt3A proteins with GFP-expressing cell lines

MDA-468 cells (ATCC® HTB-132™) were stably transfected with a GFP reporter of the canonical Wnt signaling (Addgene, #24305). These MDA-468-GFP cells were plated in 48 well plate (25000 cells/well) overnight. Then, 350 μL fresh DMEM plus 10% FBS and 50 μL L-Wnt-3a-cell-conditioned medium were added. The cells were incubated for another 18 hours before imaging the GFP expression with the EVOS^®^ FL Auto Cell Imaging System.

### Quantifying Wnt3A proteins with luciferase-expressing cell lines

MDA-468 cells (ATCC® HTB-132™) were stably transfected with a luciferase reporter of the canonical Wnt signaling (Addgene, #24308). MDA-468-luciferase cells were plated in 96 well plate (5000 cells/well) in DMEM medium plus 10% FBS for 24 hours. Then, 150 μL fresh DMEM plus 10% FBS and 50 μL L-Wnt-3a-cell-conditioned medium were added and incubated for another 18 hours. The medium was then removed, and cells were washed with PBS once before 200 μL cell lysis buffer was added and incubated for 10 minutes at room temperature. 50 μL cell lysates, 50 μL luciferase substrate A and 50 μL luciferase substrate B from the luciferase assay kit were mixed and the light signals were immediately read with a luminometer. The light intensity was calibrated to a standard curve to calculate the amount of Wnt3A proteins.

### Statistical analysis

The data were presented as the mean ± S.D.. We employed an unpaired t-test to compare two groups and one-way ANOVA to compare more than two groups. P<0.05 was considered statistically significant.

## Results

### Two-dimensional (2D) adherent culturing of L-Wnt-3a-cells

**Fig 2:**
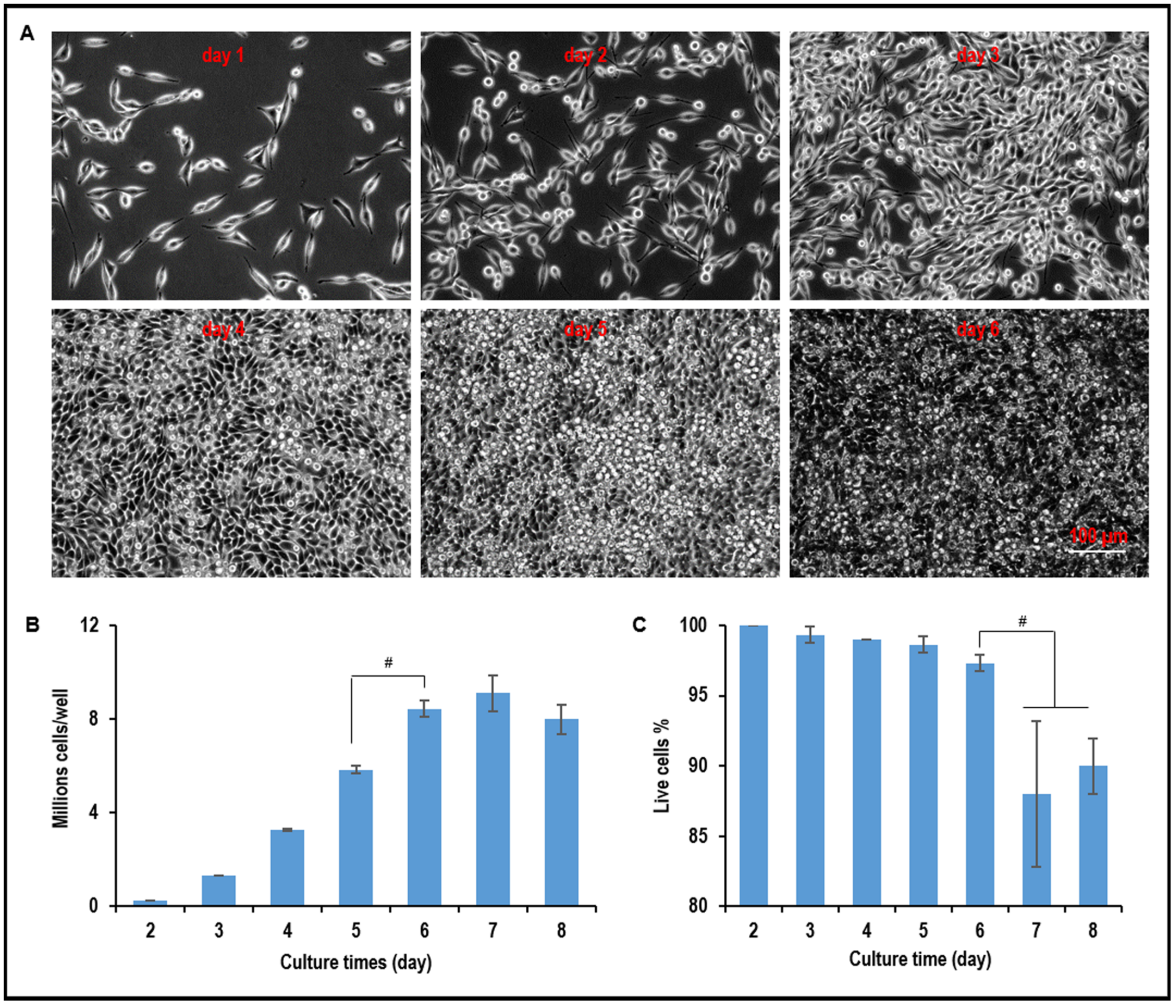
Two dimensional (2D) adherent culture of L-Wnt-3a-cells. (A) Phase images of L-Wnt-3a-cells in 2D culture from day 1 to day 6. (B) The yield and (C) cell viability from day 2 to day 8. Error bars represent the standard deviation (n = 3). #: P<0.05.

When L-Wnt-3a-cells were plated in a 6-well plate, they quickly attached to the surface, proliferated and became confluent by day 4. Multiple layers of cells were observed, and significant numbers of cells detached from the plate on and after day 5 (Fig 2A). When plated at 1x10^5^ cells per well, 3.3x10^6^, 5.8x10^6^, 8.4x10^6^, 9.2x10^6^, and 8x10^6^ cells were produced on day 4, 5, 6, 7, 8, respectively (Fig 2B). The cell viability decreased significantly and there was no further increase in cell numbers after day 6 (Fig 2C). These results show that L-Wnt-3a-cells can be cultured for about 6 days without significant cell loss in 2D adherent culturing.

### Three-dimensional (3D) suspension culturing of L-Wnt-3a-cells

**Fig 3:**
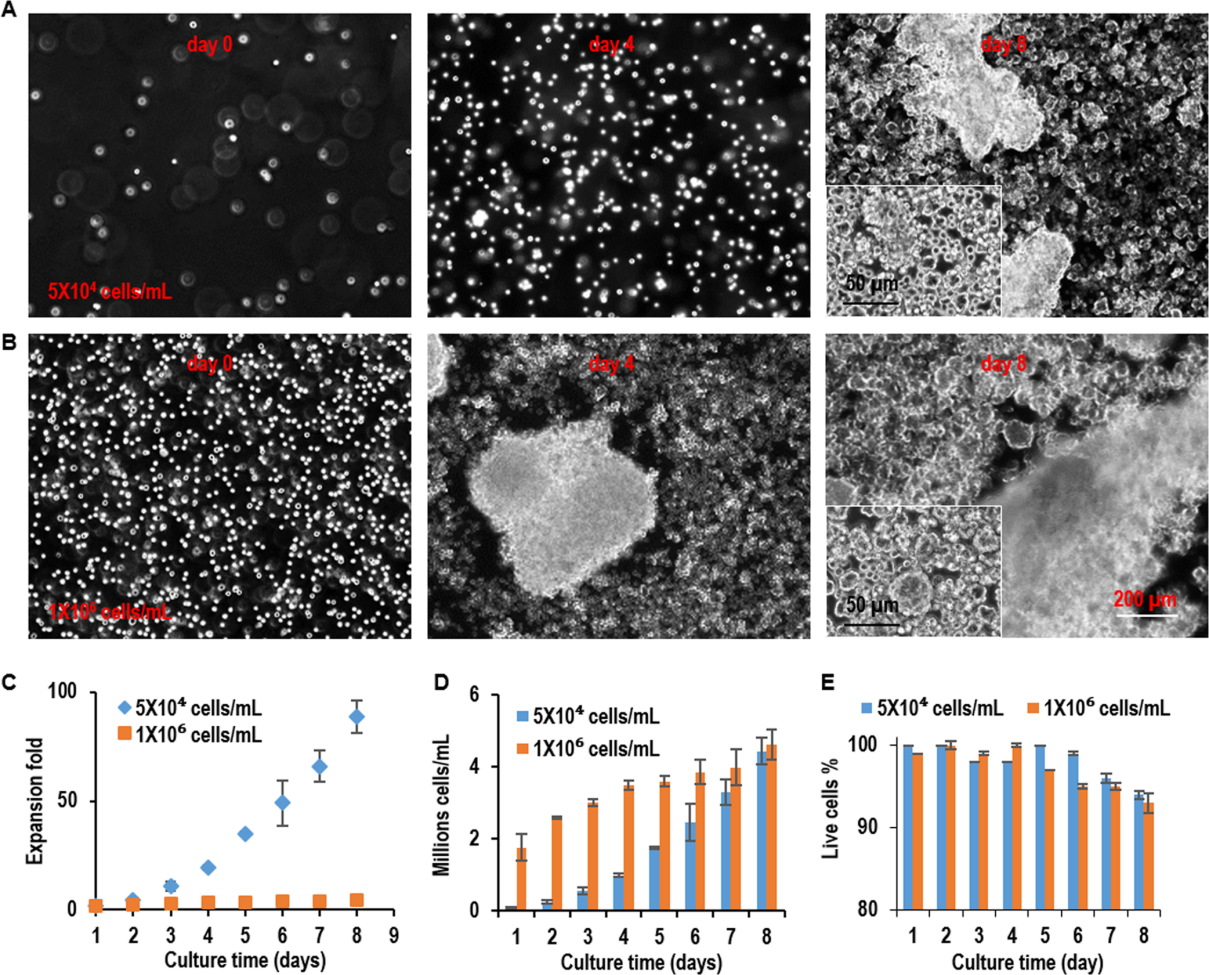
3D suspension culture of L-Wnt-3a-cells. L-Wnt-3a-cells were suspended in the low-attachment 6-well plate that was placed on a shaker at 60 rotations per minute (rpm) at low density (5x10^4^ cells/mL) and high density (1x10^6^ cells/mL). (A, B) Phase images of cells on day 0, day 4 and day 8. (C, D, E) The expansion fold, volumetric yield, and cell viability from day 1 to day 8 of the culture. Error bars represent the standard deviation (n = 3). #: P<0.05.

3D suspension culturing is widely used to scale up the production of cells and proteins [2,12,25,26]. Whether L-Wnt-3a-cells can be cultured in suspension is unknown and has not been studied. We suspended and cultured L-Wnt-3a-cells in the low-attachment 6-well plate that was placed on a shaker at 60 rotations per minute (rpm). To study the effect of seeding density, cells were seeded at low (5x10^4^ cells/mL) or medium density (1x10^6^ cells/mL) (Fig 3). At low seeding density, cells grew as single cells in the first four days. However, apparent cellular aggregates were found on day 8 (Fig 3A). Cells expanded ~4.7, 20, 49 and 89-fold, yielding ~0.2, 1, 2.5, and 4.4x10^6^ cell/mL on day 2, 4, 6 and 8, respectively (Fig 3C and 3D). At high seeding density, cell aggregation become apparent on day 4 and became severe on day 8 (Fig 3B). Severe cellular aggregation is highly unwanted for cell culture since cell aggregates larger than 400 μm lead to impaired mass transport, cell growth, viability and phenotypes [27,28]. Cells expanded about ~2.6, 3.5, 3.9 and 4.6-fold expansion, generating ~2.6, 3.5, 3.9, and 4.6x10^6^ cell/mL on day 2, 4, 6 and 8, respectively (Fig 3C and 3D). The cell viability decreased significantly after day 7 at low seeding density and day 5 at high seeding density, respectively (Fig 3E). These results show L-Wnt-3a-cells can be effectively cultured in suspension with a maximal yield around 4.6x10^6^ cell/mL.

### Culturing L-Wnt-3a-cells in 3D PNIPAAm-PEG hydrogels

**Fig 4:**
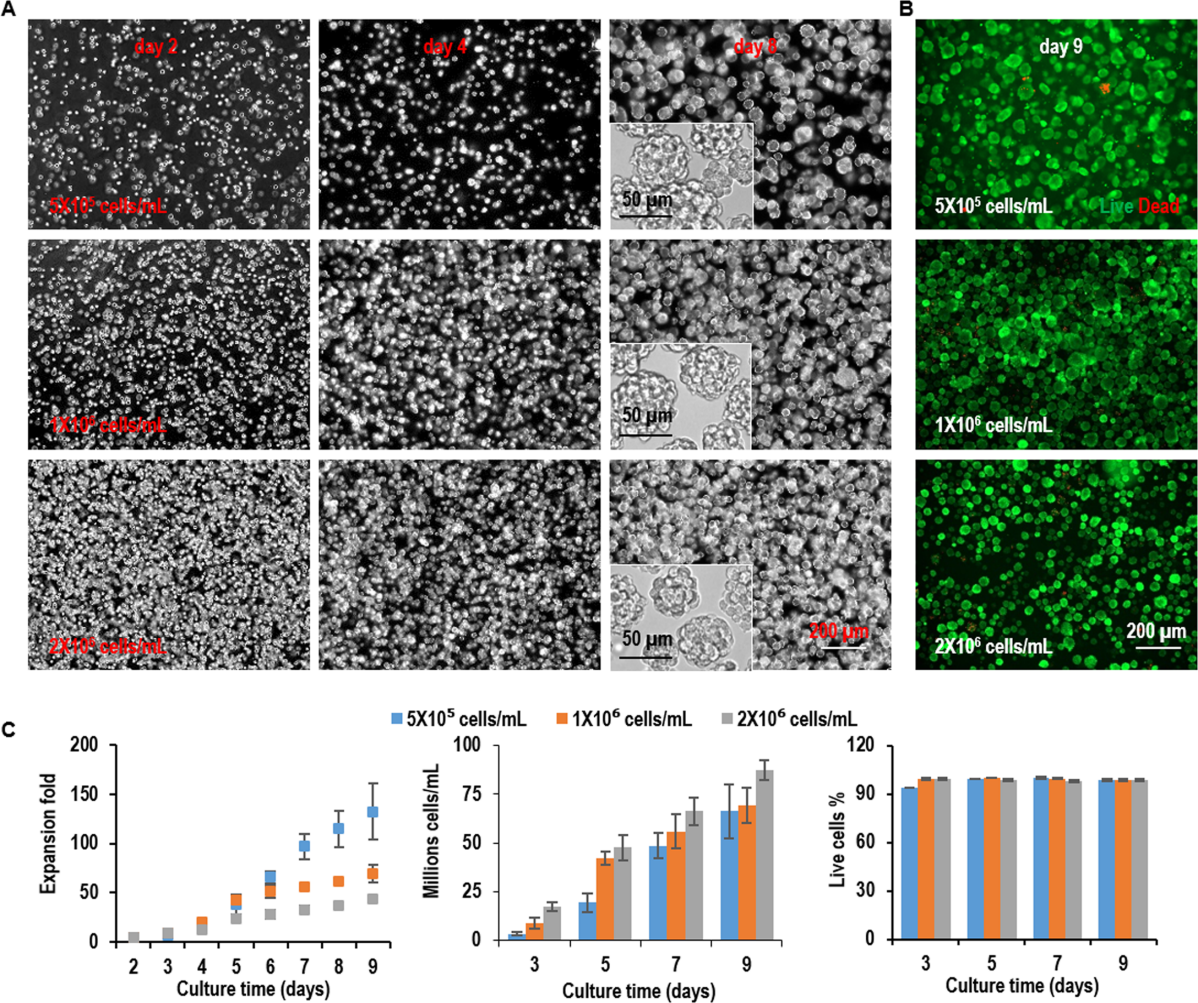
Culture L-Wnt-3a-cells in 3D PNIPAAm-PEG hydrogels (Passage 1). L-Wnt-3a-cells were cultured in the hydrogels with three seeding densities: low (5x10^5^ cells/mL), medium (1x10^6^ cells/mL), and high density (2x10^6^ cells/mL). (A) Phase images of the passage 1 cells in the hydrogels on day 2, 4, 6 and 8 of the culture. (B) Live (green) dead (red) staining of day 9 cells in the hydrogels in passage 1. (C) The expansion fold, volumetric yield and cell viability along a 9-day culture in passage 1. Error bars represent the standard deviation (n = 3).

L-Wnt-3a-cells were cultured in the hydrogel with three seeding densities, low (5x10^5^ cells/mL), medium (1x10^6^ cells/mL), and high density (2x10^6^ cells/mL). For all seeding densities, single L-Wnt-3a-cells proliferated and grew into spheroids with uniform size. The spheroid size was bigger at lower seeding density (Fig 4A). Live dead staining detected very few dead cells during the entire culture (Fig 4B). When seeded at 5x10^5^ cells/mL, cells expanded 6.9, 38.7, 97.1, and 132.5-fold to yield 3.4x10^6^, 1.9x10^7^, 4.9x10^7^, and 6.6x10^7^ cells/mL on day 3, 5, 7, and 9, respectively. When seeded at 1x10^6^ cells/mL, cells expanded 8.8, 42.3, 55.9, and 69.4-fold to yield 8.8x10^6^, 4.2x10^7^, 5.6x10^7^, and 6.9x10^7^ cells/mL on day 3, 5, 7, and 9, respectively. When seeded at 2x10^6^ cells/mL, cells expanded 8.7, 23.9, 33.2, and 43.6-fold to yield 1.7x10^7^, 4.8x10^7^, 6.6x10^7^, and 8.7x10^7^ cells/mL on day 3, 5, 7, and 9, respectively. For all seeding densities, cell viability was above 98% (Fig 4C). These results show L-Wnt-3a-cells can be cultured in the thermoreversible PNIPAAm-PEG hydrogel at a high growth rate and volumetric yield. The volumetric yield is about 10 to 20 times of the 3D suspension culturing (Fig 3).

**Fig 5:**
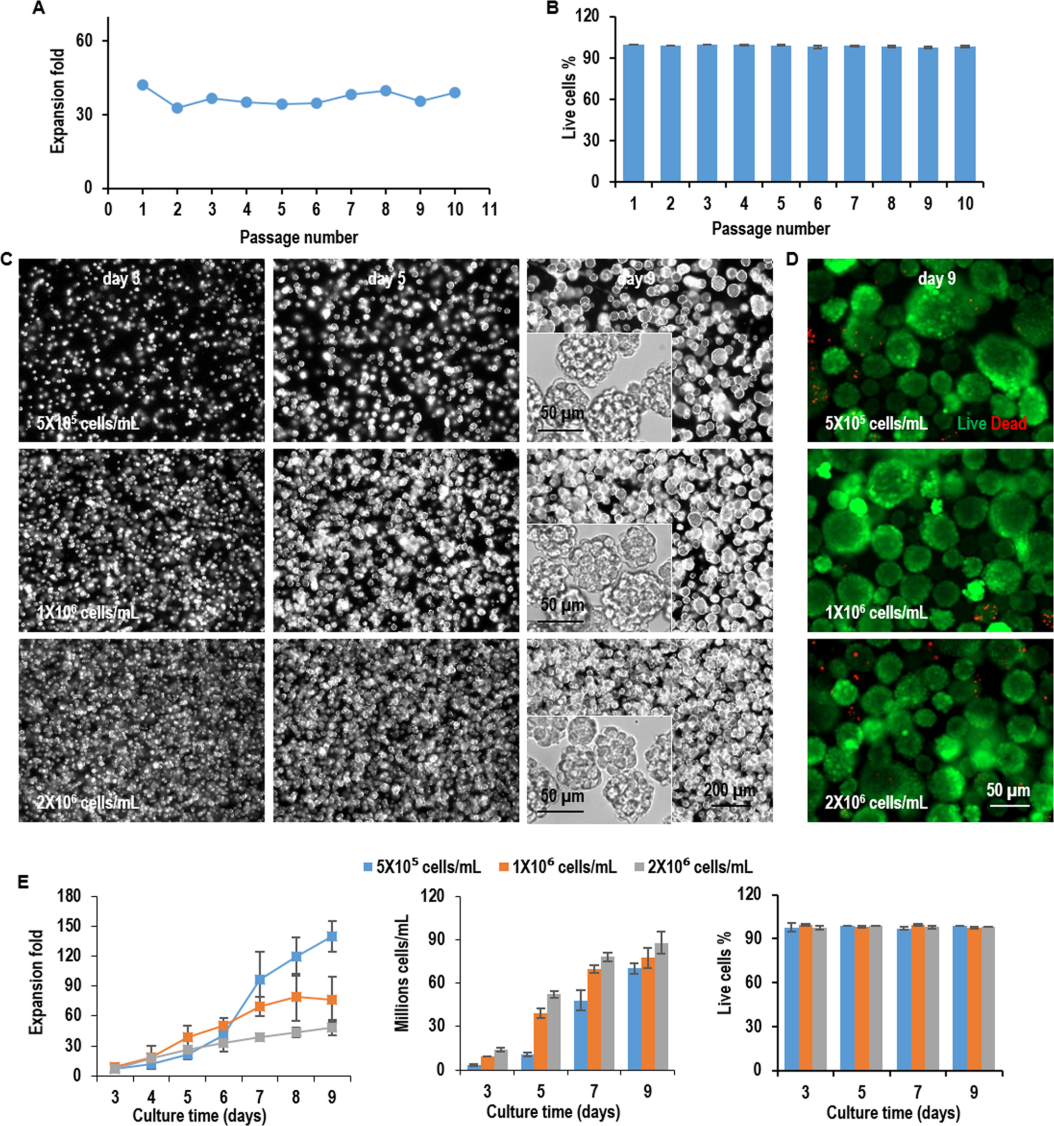
Long-term culture of L-Wnt-3a-cells in 3D PNIPAAm-PEG hydrogels. (A, B) The expansion fold and cell viability of L-Wnt-3a-cells cultured in the hydrogels from passage 1 to passage 10. Cells were passaged every 5 days. (C) Phase images of cells in the hydrogels on day 3, 5, 7, 9 at passage 10. (D) Live (green) dead (red) staining of day 9 cells in the hydrogels at passage 10. (E) The expansion fold, volumetric yield and cell viability along a 9-day culture at passage 10. Error bars represent the standard deviation (n = 3).

Next, we studied whether L-Wnt-3a-cells could be cultured in the hydrogel for long-term. During a 10-passage culture in the hydrogel, when seeded at 1x10^6^ cells/mL, L-Wnt-3a-cells consistently expanded ~37-fold per passage per 5 days with cell viability >98% (Fig 5A and 5B). To assess whether the long-term culture altered the cell phenotype, we re-evaluated the growth kinetics of L-Wnt-3a-cells at passage 10. We found the cell morphologies, growth rate, cell density and viability were very similar to these at passage 1, indicating the hydrogel supports long-term culturing of L-Wnt-3a-cells without significantly altering their phenotype (Fig 5C–5E).

### Wnt3A protein production with L Wnt-3A cells

**Fig 6:**
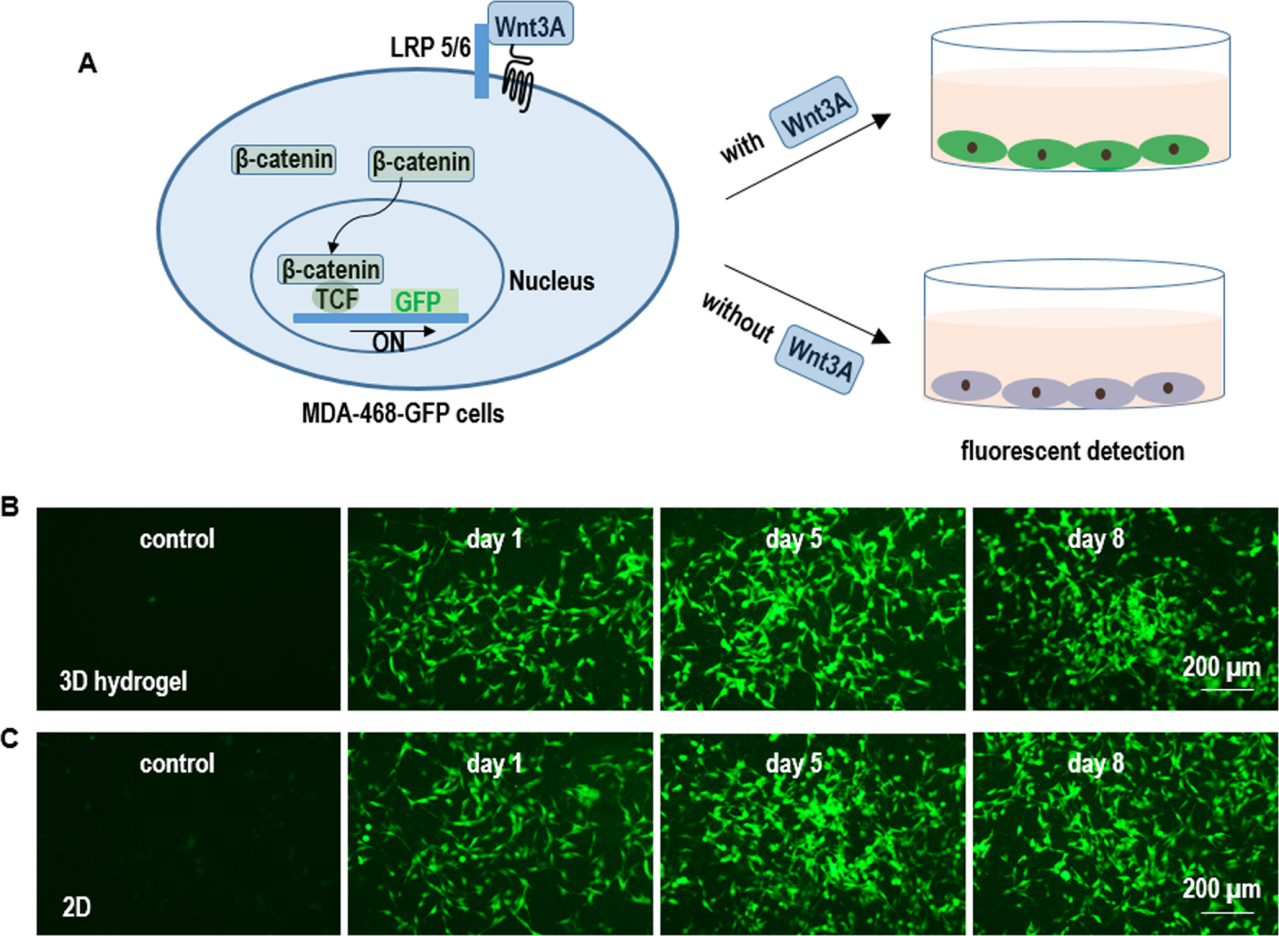
Qualitative detection of Wnt3A protein production in 2D adherent & 3D hydrogel cultures. (A) MDA-468 cells (ATCC HTB-132^TM^) were stably transfected with a GFP reporter for the canonical Wnt signaling (Addgene, #24305). In the presence of biologically active Wnt3A proteins, the MDA-468-GFP cells express GFP proteins. (B, C) MDA-468-GFP cells expressed high level of GFPs when treated with L-Wnt-3a-cells-conditioned medium from both the 2D adherent & 3D hydrogel cultures. The control medium induced no GFP expression.

To analyze whether the cultured L-Wnt-3a-cells secreted biologically active Wnt3A proteins, we generated MDA-468 cells that stably expressed a GFP reporter for the canonical Wnt signaling [29]. When active Wnt3As binding to their receptors at the cell surface, the β-catenin proteins in the cytosol enter the nucleus, form a complex with TCF proteins and initiate the GFP reporter expression (Fig 6A). L-Wnt-3a-cells in both the adherent culturing and 3D hydrogels secreted active Wnt3A proteins as shown by the high level of GFPs in MDA-468-GFP cells treated with the conditioned medium (Fig 6B and 6C).

**Fig 7:**
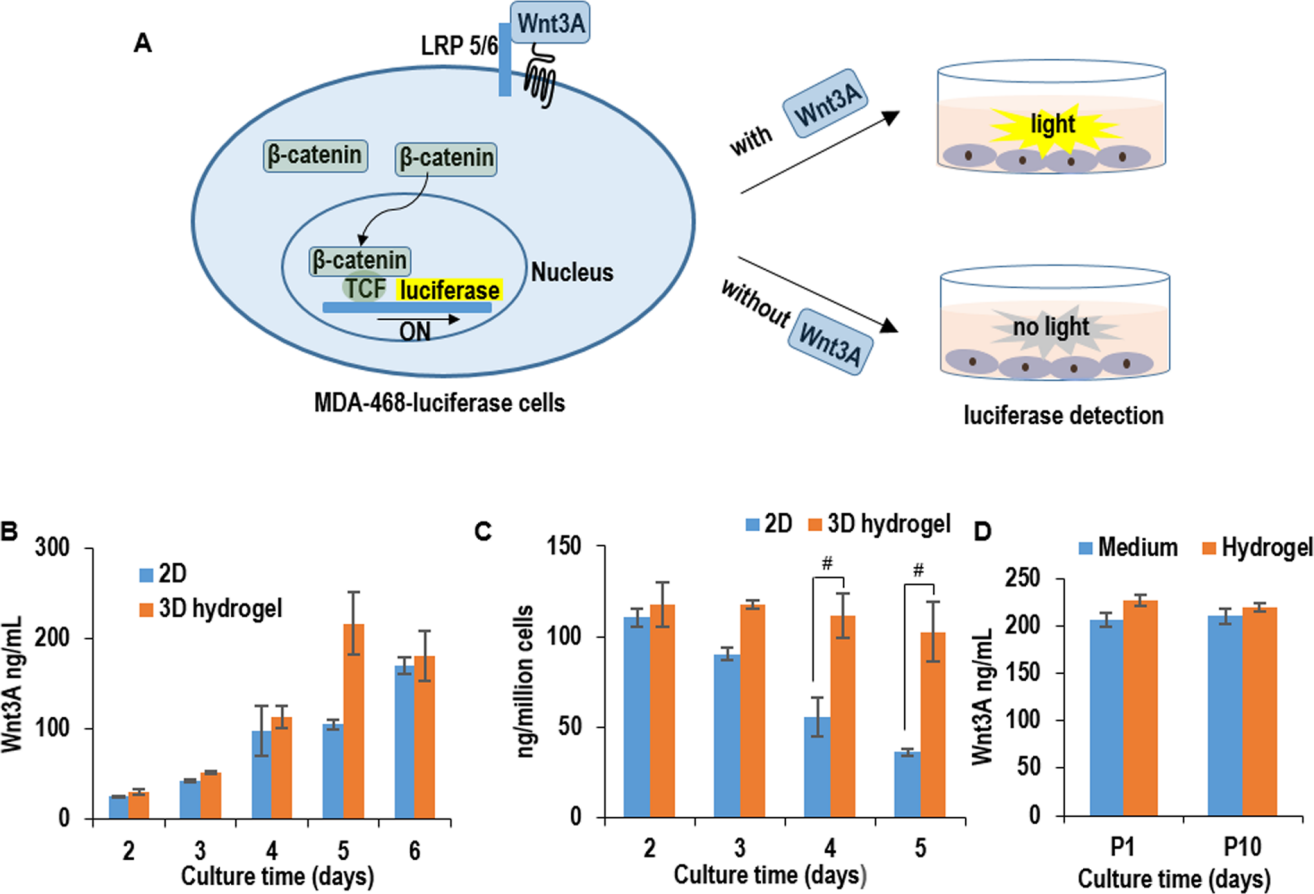
Quantitative detection of Wnt3A protein production in 2D adherent & 3D hydrogel cultures. (A) MDA-468 cells (ATCC HTB-132^TM^) were stably transfected were with a luciferase reporter for the canonical Wnt signaling (Addgene, #24308). In the presence of biologically active Wnt3A proteins, the MDA-468-luciferase cells express luciferase proteins, which can be quantified with luciferase assay. (B) The concentration of Wnt3A proteins in the conditioned medium in 2D adherent & 3D hydrogel cultures from day 2 to day 6. (C) The protein productivity (i.e. ng of Wnt3A produced per cell per day) on day 2, 3, 4, and 5 in 2D adherent & 3D hydrogel cultures. (D) The Wnt3A concentration in the conditioned medium and hydrogel scaffold on day 5 in 3D hydrogel cultures. Error bars represent the standard deviation (n = 3). #: P<0.05.

To quantify the active Wnt3A proteins in the conditioned medium, we generated MDA-468 cells that were stably transfected with a luciferase reporter of the canonical Wnt signaling [29]. We first treated these MDA-468-luciferase reporter cells with commercially-acquired Wnt3A proteins to generate a standard curve. With this standard curve, we quantified the active Wnt3A proteins in the L-Wnt-3a-cell-conditioned medium. For both adherent and 3D hydrogel culturing, Wnt3A concentration was around 25, 42, 97, 105 and 170 ng/mL in the conditioned medium on day 2, 3, 4, 5, and 6, respectively. When normalized to the cell numbers, cells produced 110, 65, 60 and 36 ng per cell per day on day 2, 3, 4 and 5 in adherent culturing, while consistently produced around 110 ng per cell per day on day 2, 3, 4 and 5 in 3D PNIPAAm-PEG hydrogels (Fig 7B and 7C). To evaluate whether the hydrogel scaffold limited the diffusion of Wnt3A proteins into the medium, we quantified the Wnt3A concentration in the conditioned medium and in the hydrogel. The concentrations were similar (e.g. ~200 ng/mL), indicating the diffusion of Wnt3A proteins in the hydrogel scaffold was very efficient (Fig 7D). We also found cells expressed a similar level of proteins at passage 1 and passage 10, indicating the long-term culturing in 3D PNIPAAm-PEG hydrogels did not reduce cells’ productivity (Fig 7D).

**Fig 8:**
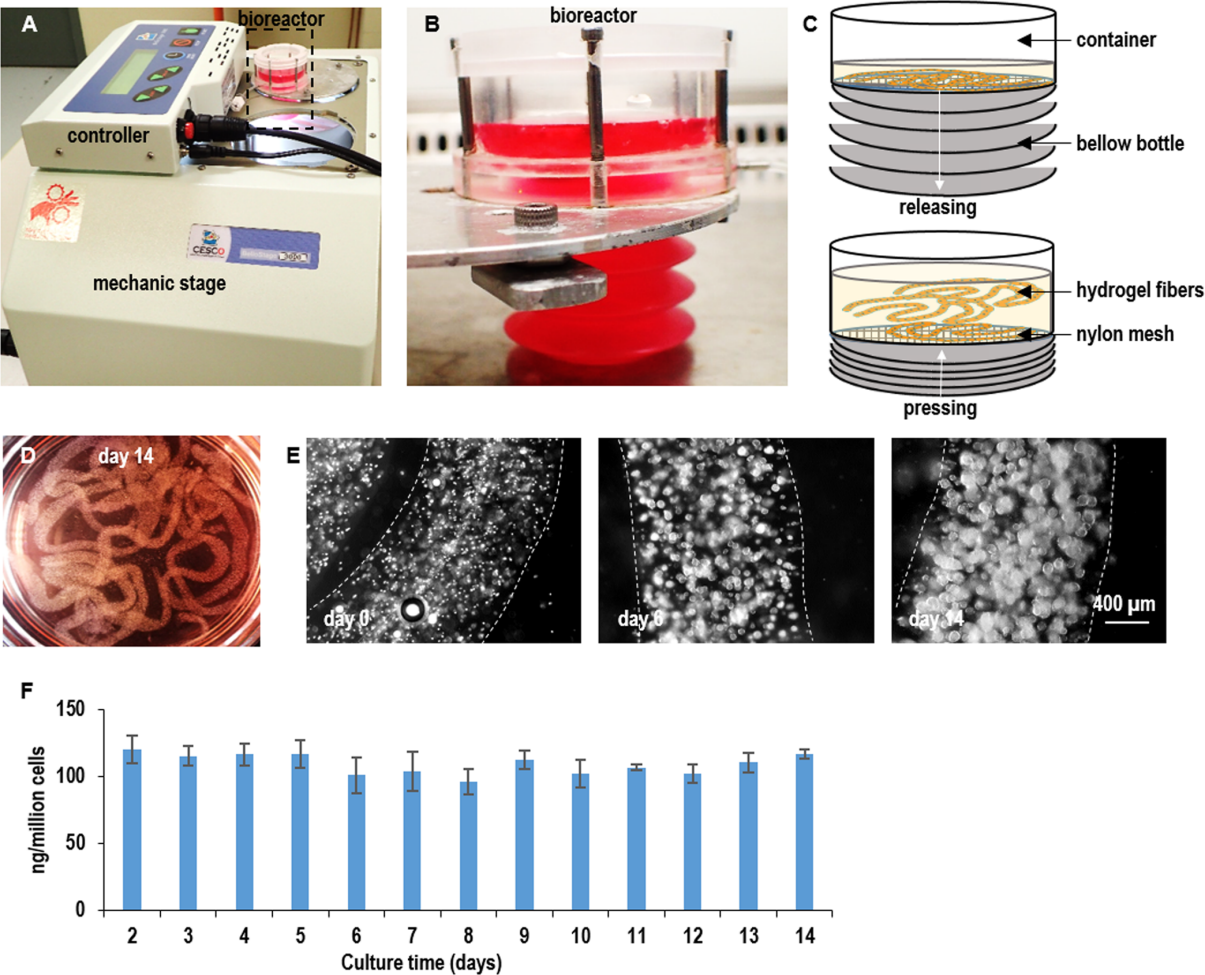
A prototype bioreactor for producing Wnt3A proteins. (A, B, C) Single L-Wnt-3a-cells were mixed with 10% PNIPAAm-PEG solution and processed into hydrogel fibers with an extruder. Hydrogel fibers with cells were suspended within a column bioreactor. The medium was stored in a plastic bottle that could be pressed to flow the medium into or released to withdraw the medium from the bioreactor. (D) Image of hydrogel fibers with cells in the bioreactor. (E) Phase images of cells in hydrogel fibers on day 0, day 6 and day 14. (F) Cells consistently expressed Wnt3a during the two-week culture. Error bars represent the standard deviation (n = 3).

We developed a prototype bioreactor to demonstrate the application of PNIPAAm-PEG hydrogels for the scalable culturing of L-Wnt-3a-cells and production of Wnt3A proteins (Fig 8A–8C). Single L-Wnt-3a-cells were mixed with 10% PNIPAAm-PEG solution and extruded into hydrogel fibers that were suspended within a bioreactor. The medium was stored in a plastic bellow bottle that could be pressed to flow the medium into or released to withdraw the medium from the bioreactor. About 7x10^7^ cells per mL of hydrogel were produced in the bioreactor (Fig 8D and 8E). Consistent Wnt3A production was achieved during a two-week culturing (Fig 8F). This prototype bioreactor can be further scaled up in the future.

## Discussion

As aforementioned, 2D adherent culturing is limited by its low yield and suspension culturing is preferred for large-scale cell culturing [2,12]. However, there are two significant challenges with suspension culturing. The first is the cellular aggregation. Mammalian cells usually have strong cell-cell interactions that make them aggregate [30,31]. Suspended cells tend to form large cell agglomerates (i.e., agglomeration). Agglomeration leads to inhomogeneity in cell aggregate size and is detrimental to cell culture [28]. For instance, the transport of nutrients, oxygen, and growth factors to, and the metabolic waste from cells located at the center of large cell agglomerates (e.g., >400 μm diameter) become insufficient, leading to slow cell growth, apoptosis, and phenotype change [27,28]. CHO cells have been genetically engineered to have the capability to grow as single cells to overcome this challenge [2,3]. Other mammalian cells such as the L-Wnt-3a-cells cannot be easily engineered and adapted to grow as single cells, and thus suffer from the cellular aggregation (Fig 3). The second challenge is the hydrodynamic stresses. Agitation is usually used to enhance the mass transport and reduce cell agglomeration in suspension culturing. However, agitation generates complicated hydrodynamic conditions including the medium flow direction, velocity, shear force, and chemical environment. These conditions vary spatially and temporally, resulting in locations (e.g. close to the vessel wall) with critical stresses that induce cell death and phenotype changes, leading to low cell viability, growth, and yield [17,28,32–38]. Again, CHO cells have been engineered and selected to be able to resist the hydrodynamic stresses and grow up to 2x10^7^ cells/mL. Other cell types are much more susceptible to the stresses and can only grow at low or moderate density (Fig 3).

The PNIPAAm-PEG hydrogel scaffolds not only provide 3D spaces for the cells, but also act as physical barriers to prevent excessive cellular agglomeration and protect cells from the hydrodynamic stresses (Fig 1). As a result, cells can grow at high viability, high growth rate, and extremely high yield even without genetic manipulations (Figs 4 and 5). The cell yield in the hydrogels is around 10 to 20 times of the suspension culturing. In addition, the protein productivity per cell per day in the hydrogel is much higher than the adherent culturing method (Fig 7).

## Conclusion

In conclusion, a simple, efficient, and scalable 3D culture system based on thermoreversible hydrogel was developed for culturing protein-producing mammalian cells with high cell viability, growth rate, volumetric yield, and protein productivity. To the best of our knowledge, this is the first report using a 3D thermoreversible hydrogel for protein production. This simple method provides a valuable tool for research laboratories and pharmaceutical industry for producing therapeutic proteins.
